# Safe and respectful? Birth attendants’ hand hygiene compliance and its determinants using nationally representative data from Kenya, Malawi and Nepal

**DOI:** 10.1186/s13756-025-01634-2

**Published:** 2025-10-21

**Authors:** Lucia Dansero, Giorgia Gon

**Affiliations:** 1 Department of Clinical and Biological Sciences, Centre for Biostatistics, Epidemiology, and Public Health, University of Turin, Orbassano, TO Italy; 2 Department of Infectious Diseases, London School of Hygiene and Tropical Medicine, London, UK

**Keywords:** Hand hygiene, Newborn health, Maternal health, Birth attendants

## Abstract

**Background:**

In low and middle-income countries (LMICs), infections acquired during childbirth contribute significantly to maternal and neonatal mortality. Hand hygiene (HH) is critical in preventing the spread of infections, yet compliance remains inadequate. This study investigates birth attendants’ HH compliance during labour, delivery, and postpartum in LMICs, using nationally representative data from Service Provision Assessments (SPAs) in Kenya, Malawi, and Nepal.

**Methods:**

We analysed 1565 observed deliveries across 517 health facilities, resulting in 3919 HH opportunities. The outcomes were hand washing or hand disinfectant use: (1) before any initial examination, (2) before aseptic procedures during labour, (3) after birth. We used descriptive statistics to assess HH compliance and multivariate multilevel mixed-effect logistic regressions to investigate determinants, accounting for facility and individual clustering.

**Findings:**

Hand hygiene compliance varied significantly across countries, with Kenya showing the lowest rates, while Malawi and Nepal had higher compliance levels. Supportive and effective communication towards pregnant women was significantly associated with an increase in HH compliance before the vaginal examination (Kenya - OR: 5.94, 95% CI 1.68-21.0; Malawi - OR: 2.19, 95% CI 1.04–4.65) and before aseptic procedures (Kenya - OR: 4.03, 95% CI 1.81–8.96; Malawi - OR: 4.01, 95% CI 1.69–9.50; Nepal - OR: 2.66, 95% CI 1.30–5.44). HH compliance during aseptic procedures during labour was also associated with recent IPC training in Malawi (OR: 3.48,95%CI 1.44–8.41) and facility infrastructure (OR: 6.14,95%CI 1.07–35.3).

**Conclusion:**

Low hand hygiene compliance during birth, especially before aseptic procedures, can lead to healthcare-associated infections with serious consequences for mothers and newborns. Future research should investigate further the association between effective communication and hand hygiene.

**Supplementary Information:**

The online version contains supplementary material available at 10.1186/s13756-025-01634-2.

## Background

Infections acquired during birth contribute to approximately 10% of maternal deaths and 11–19% of neonatal deaths worldwide, most of which occur in low and middle-income countries (LMICs) [1–4]. It has been estimated that hospital-acquired infections (HAIs) are three to twenty times more likely to occur in newborns in LMICs than in high-income countries (HICs) [5, 6]. In the last decades, there has been a considerable shift toward delivery in healthcare settings in most of LMICs, however, this has not always led to the reduction in neonatal and maternal deaths that was expected [7]. With a substantially increasing number of deliveries in healthcare facilities in LMICs, infection prevention and control (IPC) during labour, delivery, and after birth is fundamental.

Hand hygiene (HH) is one of the key IPC aspects to prevent infections. If performed appropriately, it is the single most effective and simplest procedure to reduce transmission of pathogenic microorganisms in health care [8–10]. Beyond preventing the direct spreading of pathogens, adequate HH compliance is fundamental to ensure the safety of other surfaces and equipment. The World Health Organization (WHO) define five moments for HH during patient care – before touching a patient, before a procedure, after the procedure or body fluids exposure risk, after touching a patient, and after touching a patient’s surroundings [11]. Childbirth consists of multiple procedures resulting in more than one moment when HH should be performed. The HH moment recognised as having the highest risk of infection transmission is WHO moment 2 - before clean/aseptic tasks when there is potential contact with the patient’s mucous membranes or nonintact skin. During delivery and birth, the second moment of HH could be indicated as before and during vaginal examination or delivery and related procedures [12–14].

Poor compliance with HH could be influenced by many factors and behaviours, such as inadequate infrastructure, overcrowding, lack of staff and training, and high patient loads [11]. Given the complexity of childbirth and the extreme vulnerability of women and newborns during this time, along with the demands of conducting direct observations, collecting HH data during delivery can be challenging. Little information is available on birth attendants’ hand hygiene compliance in LMICs. A recent review found only a few studies, most of poor quality and from a small number of facilities, reporting highly variable but generally low compliance [15].

The Service Provision Assessments (SPA) are nationwide surveys providing an extensive overview of a country’s health services, including direct observations of labour and delivery services. Our study aimed to investigate the compliance with HH of birth attendants during labour, delivery, and immediately after birth and analyse its determinants using data from the SPA conducted in Kenya, Malawi, and Nepal.

## Methods

### Data sources

We used data from the SPA, a cross-sectional nationally representative survey on the delivery of health services run by the Demographic and Health Surveys (DHS) Programme.

The Kenya SPA (KSPA) of 2010, the Malawi SPA (MSPA) of 2013, and the Nepal SPA (NSPA) of 2021 were selected for the analysis as they include a specific section on direct observation of pre-labour vaginal examination, labour, normal vaginal delivery, and post-birth practices. The observation structure and the survey methods were consistent across all three countries [16–18]. Data from the delivery observations were anonymously linked to data on the facilities’ services, delivery services, water, sanitation and hygiene (WASH), and interviews of providers.

### Sample

The sample of health facilities for the KSPA and NSPA was a stratified random sample selected from a Master Facility List in Kenya and via equal probability systematic sampling with sample allocation in Nepal. Instead, the MSPA was intended as a census of all facilities on the Ministry of Health master list. In the KSPA and NSPA, some strata (e.g., hospitals) are oversampled. For the delivery observation, in over 90% of facilities, all labour and delivery clients at the facility present on the day of the survey were included in the observation.

### Variables definition

The main outcome of interest was observed HH (either hand washing with soap or the use of hand disinfectant) during labour and delivery practice. Three HH opportunities were selected from the survey as follows:

1. Whether or not the birth attendant washes hands with water and soap or uses hand disinfectant before any initial vaginal examination of the client.
2. Whether or not the birth attendant washes hands with water and soap or uses hand disinfectant before aseptic procedures during labour.
3. Whether or not the birth attendant washes hands with water and soap or uses hand disinfectant immediately after birth.

The first two HH opportunities can be classified as WHO HH moment 2 (before clean/aseptic procedures), whereas the third opportunity lies between WHO moment 4 and 5 (after touching a patient/patient’s surroundings) [11].

To define the presence and usage of gloves, we used three variables collected in the SPA: the presence of gloves in the delivery room, glove use before vaginal examinations, and glove use during labour. However, these data were not available for all countries.

To explore the potential risk factors for HH delivery practices, we selected variables previously associated with HH compliance in other studies or deemed relevant in the existing literature.

We included region, facility type, and managing authority to account for facility structure and location. To assess resource availability, we constructed a variable measuring the presence of water, soap, and hand disinfectant in the delivery room on the survey day [11, 19–22]. Personal characteristics of birth attendants included sex [23], years of experience in delivery practices, number of deliveries in the past six months, recent training in Infection Prevention and Control or delivery care [22, 24], and the technical qualification – data not shown - (specialist/midwife vs. nurse in Malawi and Kenya; specialist/obstetrician vs. nurse/nurse midwife in Nepal). While qualification predicts HH compliance in other studies [11, 19, 25], our sample was skewed toward one or two cadres (nurse/midwife), so we treated it as an a priori confounder rather than analysing its effect. To assess birth attendants’ behaviour and quality of care, we created a variable called “effective communication”, selecting all available variables related to providers’ behaviour towards patients during the pre-delivery examination, labour, and deliverycommunication [26–28] Since not all women were observed for the entire duration of delivery, we created two separate variables—one for each outcome. For the first outcome (hand hygiene before vaginal examination), we combined “respectfully greeting the woman” and “asking the woman (and support person) if she has any questions.” For the second outcome (hand hygiene during labour before aseptic procedures), we combined “explaining all procedures in labour to the woman and her support person before proceeding” and “supporting the mother throughout labour in a friendly manner.”

### Ethics

For the Service Provision Assessments, the Institutional Review Board (IRB) of the country where the survey takes place ensures that the survey complies with the country’s regulations. ICF International IRB ensures it complies with the U.S. Department of Health and Human Services regulations for the protection of human subjects. For more information, please refer to: http://dhsprogram.com/What-We-Do/Protecting-the-Privacy-of-DHS-Survey-Respondents.cfm.

### Statistical analysis

We used descriptive statistics to estimate the levels of HH compliance by outcome, the availability of water and soap, hand disinfectant, and the availability and usage of gloves in the delivery room by country. We also estimated individual compliance - HH performed over the number of opportunities – of birth attendants that were observed during at least three HH opportunities. Afterwards, a multivariate multilevel mixed-effect logistic regression was conducted to assess what factors (introduced in the model as fixed effects) are associated with hand hygiene compliance for the first two outcomes of hand hygiene – HH before any initial vaginal examination and HH during labour specifically because the compliance to HH opportunities performed “before” a procedure/patient tends to be lower than those performed “after” and hence more insights about their drivers can inform programmatic interventions. Provider and facility were used as the two levels of clustering. We then calculated the intracluster correlation coefficients (ICCs) to estimate within and between individual and facility variation in hand hygiene [29]. This was done on providers with at least three hand hygiene opportunities observed. Sampling weights were applied to both descriptive and analytical statistics to account for the survey characteristics. All analyses were performed on STATA17.

## Results

The observations of labour and delivery of the SPAs were conducted in 517 health facilities, with 170 facilities in Kenya, 222 in Malawi, and 125 in Nepal. Overall, the dataset comprised the observation of 1565 deliveries (620 in Kenya, 474 in Malawi, and 471 in Nepal) conducted by a total of 889 (266 in Nepal, 292 in Malawi, and 331 in Kenya), resulting in 3919 HH opportunities.

**Fig. 1:**
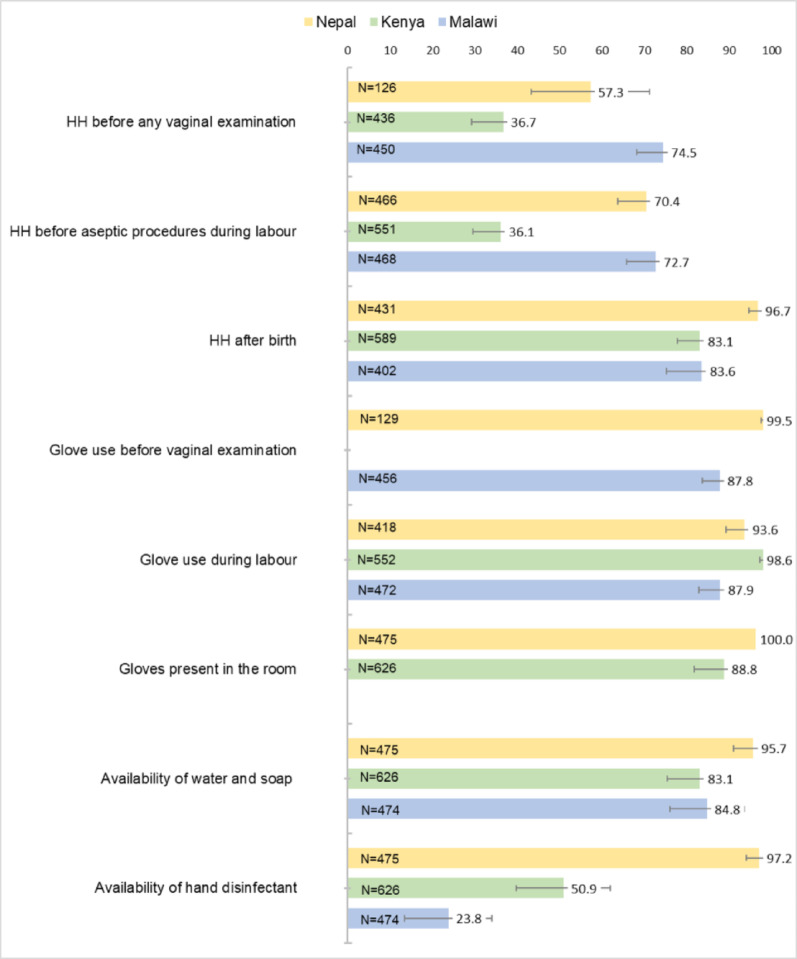
Hand hygiene compliance, glove use, and availability of equipment by country

HH compliance varies substantially by country and by outcome. As illustrated in Fig. 1, Kenya exhibits the lowest compliance across all three HH opportunities with 36.7% (95% CI 29.4–44.5) before any initial vaginal examination, 36.1% (95% CI 29.8–42.9) before aseptic procedures during labour, and 83.1% (95% CI 77.3–87.6) after birth. Malawi and Nepal have higher compliance with, respectively, 74.5% (95% CI 67.7–80.3) and 57.3% (95% CI 42.8–70.6) for the first HH outcome; 72.7% (95% CI 65.9–79.5) and 70.4% (95% CI 63.4–76.6) for the second; and 83.6% (95% CI 73.6–90.3) e 96.7% (95% CI 94.3–98.2) after birth. Overall, the third opportunity of HH considered in this study (performed after birth) has the highest compliance across all countries (Fig. 1). Compliance with glove use before vaginal examination and during labour is consistently high in all countries, ranging from 87.8 to 99.5. Additionally, gloves, water, and soap are widely available in the delivery room. Hand disinfectant is mostly present in Nepal (97.2%), whereas, in Malawi, only 23.8% (CI 13.5–34.1) of delivery rooms are equipped with hand disinfectant, and 50.9% in Kenya (95% CI 39.8–62.0).

Table 1 reports the adjusted Odds Ratios (ORs) for the association between compliance with HH before vaginal examination and the characteristics of health facilities and birth attendants included in the SPAs. Birth attendants who demonstrated supportive and effective communication towards pregnant women are significantly more likely to comply with HH before vaginal examination in both countries (Kenya - OR: 5.94, 95% CI 1.68–21.0; Malawi-OR: 2.19, 95% CI:1.04–4.65). Birth attendants’ HH compliance before vaginal examination is slightly associated with increased years of experience in delivery care in Kenya. The Kenya SPA is the only survey that included a question on the provider’s number of years of experience in conducting deliveries. No additional variables show a significant association with HH compliance before vaginal examination.

**Table 1:** Associations between hand hygiene compliance before initial vaginal examination and facility and health worker characteristics

	Kenya					Malawi				
	Total (*n* = 436)	(%)	Compliance (%)	OR (95%CI) (*n* = 411) *	*p*-value	Total (*n* = 450)	(%)	Compliance (%)	OR (95%CI) (*n* = 419) *	*p*-value
Gender										
Male	84	(19.4)	28 (33.3)	0.93 (0.29–2.99)	0.915	91	(19.1)	64 (70.3)	1.07 (0.40–2.83)	0.869
Female	347	(79.6)	131 (37.8)	1.00 (ref)		359	(80.9)	274 (75.5)	1.00 (ref)	
Training in IPC										
None	201	(46.1)	86 (42.8)	1.00 (ref)		164	(36.4)	110 (66.7)	1.00 (ref)	
Yes, in the past year	87	(20.2)	17 (19.5)	0.37 (0.10–1.35)	0.131	125	(27.9)	98 (77.2)	1.73 (0.73–4.11)	0.206
Yes, in the past 2–3 years	113	(25.9)	47 (41.6)	0.90 (0.29–2.81)	0.861	128	(28.4)	100 (78.1)	1.57 (0.72–3.93)	0.334
Missing	34	(7.8)	10 (29.4)			33	(7.3)	30 (88.2)	–	
Delivery training										
No	169	(38.9)	66 (39.1)	1.00 (ref)		169	(37.6)	121 (70.8)	1.00 (ref)	
Yes	231	(53.0)	84 (36.4)	1.36 (0.49–3.76)	0.546	248	(55.1)	186 (74.4)	1.61 (0.68–3.75)	0.271
Missing	36	(8.1)	10 (27.8)			33	(7.3)	31 (93.9)	-	
Effective communication										
No	294	(67.4)	82 (27.9)	1.00 (ref)		307	(68.2)	230 (74.0)	1.00 (ref)	
Yes	142	(32.6)	78 (54.9)	5.94 (1.68-21.0)	0.006	140	(31.1)	106 (75.7)	2.19 (1.04–4.65)	0.040
Missing						3	(0.7)	2 (66.7)		
Facility type										
Hospital	363	(83.3)	124 (34.2)	1.00 (ref)		319	(70.9)	250 (77.9)	1.00 (ref)	
Health centre /maternity/clinic	73	(16.7)	36 (49.3)	2.31 (0.30–17.6)	0.414	131	(29.1)	88 (66.2)	0.61 (0.21–1.73)	0.368
Managing authority										
Government	339	(77.8)	113 (33.3)	1.00 (ref)		360	(80.1)	273 (75.2)	1.00 (ref)	
Private/NGO	97	(22.2)	47 (48.5)	2.87 (0.94–8.73)	0.062	90	(19.9)	65 (71.4)	1.16 (0.40–3.36)	0.781
Water and soap/hand disinfectant										
None	55	(12.7)	14 (25.5)	1.00 (ref)		53	(11.8)	42 (79.2)	1.00 (ref)	
Only one	182	(41.8)	51 (28.0)	0.63 (0.14–2.99)	0.564	302	(67.2)	226 (73.6)	0.80 (0.16–3.98)	0.786
Both	199	(45.5)	95 (47.7)	1.91 (0.45–8.01)	0.439	95	(21.0)	70 (74.5)	0.52 (0.14–1.85)	0.313
Number of deliveries °	412	85.6	(115.6)	1.003 (0.99–1.01)	0.272	418	174.9	(187.6)	1.00 (0.99–1.01)	0.749
Number of years of experience °	413	7.10	(7.04)	1.07 (1.004–1.13)	0.037	-	-	-	-	-

*All models are adjusted by region and the technical qualification of the provider;

° Those variables are presented by N, mean and standard deviation (sd);

OR: Odds ratio; CI: Confidence interval; ref: reference

Table 2 shows the results for the association between compliance with HH performed during aseptic procedures during labour and the characteristics of health facilities and birth attendants. The providers in Malawi who received training in IPC within the year before the survey are more likely to comply with HH before aseptic procedures during labour compared to those who have never received training (OR: 3.48, 95% CI 1.44–8.41). However, if the training was received 2–3 years prior, there is no significant increase in compliance. This result is not observed in Kenya and Nepal, where IPC training is not significantly associated with HH compliance. Consistently with the results from the first outcome, HH compliance before aseptic procedures during labour is associated with the respectful behaviour of the birth attendant (variable “effective communication”) in Kenya (OR: 4.03, 95% CI 1.81–8.96), Malawi (OR: 4.01, 95% CI 1.69–9.50), and Nepal (OR: 2.66, 95% CI 1.30–5.44). In Nepal, birth attendants working in health centres and maternity clinics are less likely to comply with HH before aseptic procedures during labour compared to those working in hospitals (OR:0.19, 95% CI 0.05–0.78). Having running water, soap, and hand disinfectant in the delivery room is associated with HH compliance during labour in Malawi (OR:6.14, 95% CI 1.07–35.3). Moreover, as the years of experience in delivery care increase, birth attendants in Kenya are slightly more likely to perform hand hygiene before aseptic procedures during labour. No other variables are associated with HH compliance during aseptic procedures during labour.

**Table 2:** Associations between hand hygiene compliance before aseptic procedures during labour and facility and health worker characteristics

	Kenya					Malawi					Nepal				
	Total (n = 551)	(%)	Compliance (%)	OR (95% CI) (n = 520) *	*p*-value	Total (n = 474)	(%)	Compliance (%)	OR (95% CI) (n = 442) *	*p*-value	Total (n = 466)	(%)	Compliance (%)	OR (95% CI) (n = 408) *	*p*-value
Gender															
Male	87	(15.9)	27 (31.0)	0.83 (0.26–2.69)	0.769	95	(20.1)	71 (74.7)	0.66 (0.24–1.79)	0.416	9	(2.1)	4 (46.1)	0.64 (0.09–4.90)	0.673
Female	459	(83.3)	172 (37.5)	1.00 (ref)		379	(79.9)	272 (71.8)	1.00 (ref)		456	(97.9)	324 (70.9)	1.00 (ref)	
Missing	5	(0.8)	0												
Training in IPC															
None	249	(45.2)	100 (40.2)	1.00 (ref)		172	(36.3)	107 (62.2)	1.00 (ref)		359	(77.1)	256 (71.3)	1.00 (ref)	
Yes, in the past year	133	(24.1)	38 (28.6)	1.63 (0.53–5.04)	0.395	132	(27.8)	106 (80.3)	3.48 (1.44–8.41)	0.006	105	(22.6)	72 (68.4)	0.79 (0.28–2.21)	0.655
Yes, in the past 2–3 years	134	(24.3)	56 (41.8)	1.98 (0.65–6.05)	0.226	136	(28.7)	105 (77.2)	1.12 (0.39–3.23)	0.827					
Missing	35	(6.4)	5 (14.3)	–		34	(7.2)	27 (79.4)	–		2	(0.3)		-	
Delivery training															
No	206	(37.4)	81 (39.3)	1.00 (ref)		177	(37.2)	123 (69.5)	1.00 (ref)		–	–	–	–	–
Yes	308	(56.0)	111 (36.0)	0.61 (0.24–1.57)	0.731	263	(55.6)	195 (74.1)	1.97 (0.88–4.38)	0.095	–	–	–	–	–
Missing	37	(6.6)	7 (18.9)			34	(7.2)	27 (79.4)	-		–	–	–	–	–
Effective communication															
No	251	(45.5)	63 (25.1)	1.00 (ref)		117	(24.7)	66 (56.4)	1.00 (ref)		208	(44.6)	129 (62.0)	1.00 (ref)	
Yes	297	(53.9)	135 (45.5)	4.03 (1.81–8.96)	0.001	355	(75.0)	277 (78.0)	4.01 (1.69–9.50)	0.002	212	(45.5)	160 (75.4)	2.66 (1.30–5.44)	0.008
Missing	3	(0.6)	1 (33.3)			2	(0.3)	2 (100.0)			46	(9.9)	39 (85.3)	–	
Facility type															
Hospital	461	(83.6)	158 (34.3)	1.00 (ref)		335	(70.7)	246 (73.4)	1.00 (ref)		324	(69.6)	241 (74.3)	1.00 (ref)	
Health centre /maternity/clinic	90	(16.4)	41 (45.6)	1.47 (0.36–6.11)	0.591	139	(29.3)	99 (71.2)	1.15 (0.39–3.34)	0.934	142	(30.4)	87 (61.4)	0.19 (0.05–0.78)	0.022
Managing authority															
Government	422	(76.6)	137 (32.5)	1.00 (ref)		380	(80.2)	275 (72.4)	1.00 (ref)		369	(79.2)	262 (71.2)	1.00 (ref)	
Private/NGO	128	(23.4)	62 (48.4)	2.78 (0.91–8.54)	0.073	94	(19.8)	70 (74.5)	0.58 (0.20–1.71)	0.324	87	(20.8)	66 (67.7)	4.58 (0.87–24.1)	0.072
Water and soap/hand disinfectant															
None	62	(11.2)	19 (30.6)	1.00 (ref)		55	(11.9)	41 (74.5)	1.00 (ref)		66	(14.1)	36 (55.3)	1.00 (ref)	
Only one	222	(40.4)	54 (24.3)	0.35 (0.08–1.56)	0.169	316	(67.2)	232 (73.4)	3.19 (0.8–12.5)	0.095	–	–	–	–	–
Both	267	(48.4)	126 (47.2)	1.90 (0.45–8.00)	0.378	95	(20.9)	72 (75.8)	6.14 (1.07–35.3)	0.042	395	(84.8)	288 (72.3)	1.89 (0.75–4.79)	0.173
Missing											5	(1.1)	4 (71.5)		
Number of deliveries °	525	84.8	(113.2)	1.00 (0.99–1.01)	0.379	443	175.2	(186.1)	0.99 (0.99–1.00)	0.183	463	113.2	(14.7)	0.99 (0.994–1.00)	0.185
Number of years of experience °	526	6.9	(7.0)	1.06 (1.02–1.13)	0.044	–	–	–	–	–	–	–	–	–	–

*All models are adjusted by region and the technical qualification of the provider;

° Those variables are presented by N, mean and standard deviation (sd);

OR: Odds ratio; CI: Confidence interval; ref: reference

**Fig. 2:**
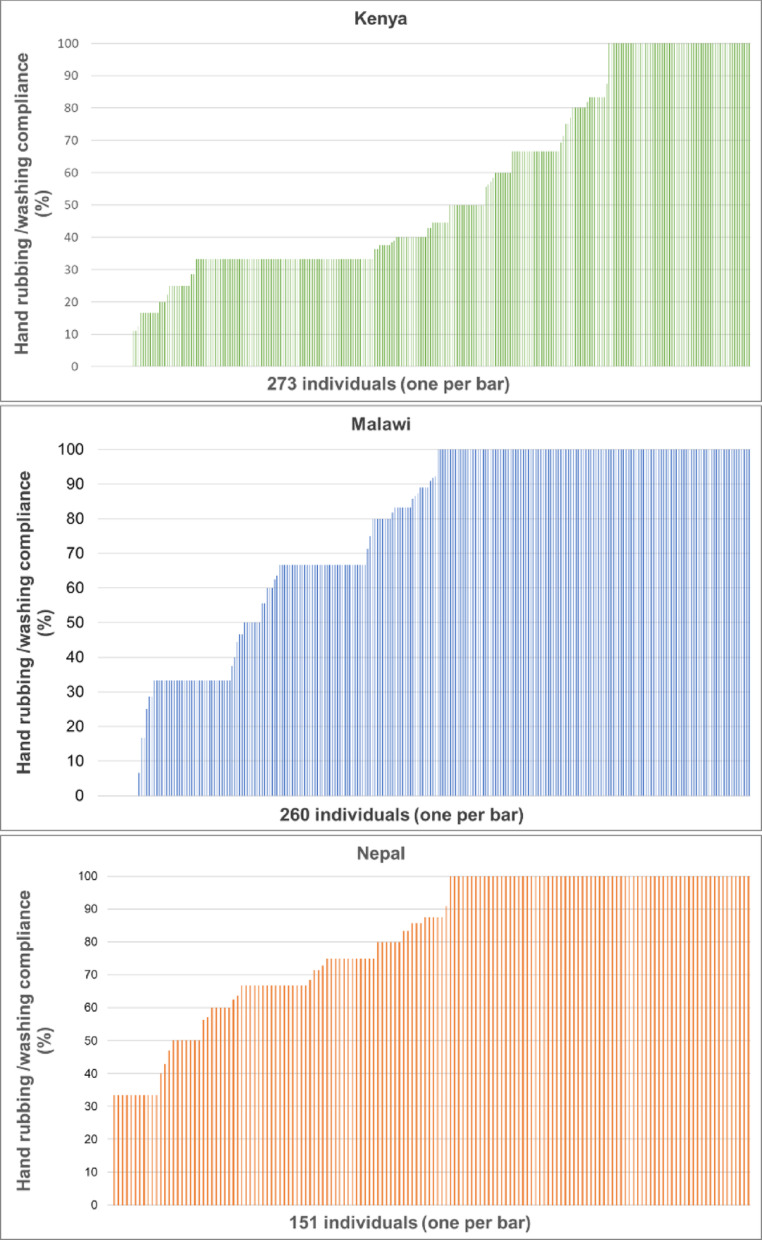
Compliance with hand rubbing/washing among individuals itieswith at least three HH opportun

We looked at individual-level compliance for those providers with at least 3 HH opportunities being observed. Overall, there are very few positive deviants across the three countries (i.e. those who are at 100% compliance), most individuals alternate between following the guidelines and not (Fig. 2).

In Kenya, the ICC for HH before vaginal examination shows moderate facility-level clustering (20%) and strong provider-level clustering (44%), as shown in Table 3. For HH before aseptic procedures, 50% of the variance is due to providers, with minimal facility-level variance (2%). In Nepal, moderate clustering occurs at both levels, with 22% of the variance before aseptic procedures attributed to providers and 14% to facilities. In Malawi, the variance attributable to facilities is negligible, so only the ICC for facilities was estimated, finding 58% for the first outcome and 61% for the second.

**Table 3:** ICC calculations for the first two opportunities of HH using crude and adjusted models

Country	Level	Crude model				Adjusted model *			
		σ^2^	ICC	SE	95% CI	σ^2^	ICC	SE	95% CI
Outcome 1: hand hygiene before vaginal examination									
Kenya	Facility	10.22	0.67	0.082	0.50–0.81	2.30	0.20	0.186	0.02–0.71
	Provider|facility	2.09	0.73	0.081	0.54–0.85	3.11	0.44	0.44	0.20–0.72
Malawi	Facility	4.29	0.57	0.099	0.37–0.74	4.59	0.58	0.106	0.37–0.76
	Provider|facility	6.67e-33	–	–	–	7.37e-33	–	–	–
Outcome 2: hand hygiene before aseptic procedures during labour									
Kenya	Facility	8.86	0.65	0.079	0.48–0.79	0.13	0.02	0.287	2.28e-15–1
	Provider|facility	1.46	0.76	0.064	0.61–0.86	3.19	0.50	0.118	0.28–0.72
Nepal	Facility	2.96	0.46	0.124	0.24–0.70	0.59	0.14	0.150	0.01–0.65
	Provider|facility	0.06	0.48	0.125	0.25–0.71	0.35	0.22	0.145	0.05–0.59
Malawi	Facility	5.65	0.63	0.095	0.43–0.79	5.06	0.61	0.107	0.39–0.78
	Provider|facility	1.71e-33	–	–	–	1.31e-33	–	–	–

*Adjusted for: gender, training in IPC, delivery training, respectful communication, facility type, managing authority, water and soap/hand disinfectant, number of deliveries, number of years of experience, region, technical qualification of the provider. σ2: variance; ICC: intraclass correlation coefficient; SE: standard error; 95%CI: 95% confidence interval

## Discussion

In this study, we analysed a total of 1565 deliveries and 3919 HH opportunities to investigate the birth attendants’ compliance with HH during childbirth in Kenya, Malawi, and Nepal, using data from the SPA, which are nationally representative surveys including direct observations of deliveries using standardised methods. Compliance with HH before aseptic procedures (before initial vaginal examination or aseptic procedures during labour) was alarmingly low across all countries, posing a serious threat of healthcare-associated infections for mothers and newborns. Effective communication was consistently associated with higher HH compliance before aseptic procedures across countries and merits further investigation.

HH compliance was low in all three countries, particularly in Kenya. Compliance before aseptic procedures was lower than HH after birth – with nearly a third of WHO Moment 2 opportunities being missed. This type of HH opportunity is the most crucial for infection prevention because hands are in contact with the patient’s body fluids. With 140 million births occurring annually, most of which are in LMIC facilities, this represents an unacceptably high risk to women and newborns [30]. Higher percentages of HH compliance after patient contact, compared to before patient contact, have been reported in previous studies [19, 31]. The lower compliance with HH before aseptic procedures, compared to after, may be due to the greater complexity of pre-procedural guidelines—requiring hand disinfection or washing, avoiding recontamination, donning gloves, and maintaining glove sterility. In contrast, post-procedure hygiene may be more strongly driven by perceived emphasis on self-protection, whereas pre-procedure actions are more directly linked to patient safety.

A recent systematic review on HH in different LMICs reported compliance before aseptic procedures ranging from 1 to 38% [15]. Overall, our findings of low compliance with HH before aseptic procedures are consistent with other studies in LMICs, especially the results from Kenya [12, 31, 32]. The higher compliance we observed in Malawi may be explained by differences in the observation tools used, compared to those in the systematic review. While many of the included studies relied on internationally recognised observation checklists specific to HH, the SPA observation tool was designed to capture the broader labour and delivery process, of which HH is only one component. This broader focus may have contributed to the higher compliance reported.

Glove presence and use were high in all countries, with rates of 87% and above, which is consistent with findings from other studies on glove use [12, 33]. However, glove misuse is known to increase the risk of cross-contamination and therefore cannot replace HH in preventing infections, which remains a pivotal component in IPC [33, 34].

Water and soap were available in most delivery rooms (> 83%), and hand disinfectant was widely available in Nepal. In contrast, only 50.9% of delivery rooms in Kenya and 23.8% in Malawi had hand disinfectant available. While the availability of appropriate items and supplies is essential for effective hand hygiene, it is not sufficient on its own. Given the high availability of these key items, we lacked the statistical power to detect a significant association between item availability and compliance. Nonetheless, we observed a small positive effect in Malawi—where the availability of hand disinfectant was lowest—on HH compliance before aseptic procedures. Hand disinfectant, included on the WHO Essential Medicines List, is considered key to facilitating hand hygiene, as it is quicker than handwashing, does not require drying materials, and can be positioned at the point of care [35].

Nearly 40% of providers observed did not receive IPC training or delivery training, which posed substantial challenges to maintaining high standards of HH and delivery care. Training in IPC in the year before the survey was associated with an increase in HH compliance before aseptic procedures in Malawi only, while the year of experience in conducting deliveries was significant in increasing HH compliance in Kenya.

In our study, we found that birth attendants’ effective communication with patients was a consistent determinant of HH compliance before vaginal examination and before aseptic procedures during labour in Kenya, Malawi, and Nepal. Providers who spend time to introduce themselves, ask the patients if there are any questions, and explain all procedures in a friendly way tend to comply more with HH compared to providers who do not have effective communication with the patients. The provision of respectful maternity care is of fundamental importance and is recommended by the WHO [36].

We acknowledge that poor communication with patients could also result from excessive workload and overcrowding of the facility. Possibly, effective communication is also a proxy for a higher overall commitment of the provider to quality care and performing their job well; however, we did find an effect despite accounting for individual-level clustering of HH behaviour. A narrative review indicated that disrespectful care could be linked to resource shortages, poor management, inadequate infrastructures, and referral systems, as well as personal attitudes [26]. The low status of midwives in the health system hierarchy was another driver of disrespectful care [26, 37, 38].

While our study shows a strong association between effective communication and HH compliance, it is important to note that this factor alone may not be enough to ensure sustained improvements. Studies have found that persistent barriers—such as limited infrastructure, resource constraints, and hierarchical social norms—can undermine hand hygiene efforts despite training and improved communication [39–41]. These factors highlight that effective communication should be part of a broader, multifaceted strategy addressing both behavioural and structural challenges in LMIC settings.

Our findings also identify critical HH moments during labour and delivery that health facilities can prioritise for routine monitoring and quality improvement to reduce infection risks. Understanding these opportunities can help tailor interventions to local contexts and enhance respectful maternity care practices. Additionally, the ICCs reported here can assist in the design of future intervention studies by informing sample size calculations.

To our knowledge, this is the first study investigating birth attendants’ compliance with HH during birth using a large sample of direct observation of deliveries, which can be considered as nationally representative data from Kenya, Malawi, and Nepal. This is a very unique dataset as direct observation of deliveries is usually very time-consuming and expensive to run at a large scale. However, we recognise some potential limitations. Firstly, the delivery observation tool included in the SPA was not specifically designed to monitor HH but rather the wider process of delivery care, thus we were unable to investigate the quality of hand washing/rubbing. Secondly, we acknowledge that HH compliance may have been partially overestimated due to a residual Hawthorne effect, as the presence of observers during clinical care can influence provider behaviour. However, the delivery observations conducted by DHS were not exclusively focused on HH but encompassed a wide range of birth practices, which likely diluted this effect. While we cannot completely rule out the influence of observation on behaviour, prior studies suggest that such generalised observations reduce the magnitude of the Hawthorne effect compared to focused HH monitoring [42, 43]. Thirdly, we recognise that the data from Kenya (2010) and Malawi (2013) are not recent, and HH compliance may have changed since then. However, these are the only publicly available nationally representative datasets that include direct observations of labour and delivery care, which makes them uniquely valuable for studying hygiene practices around childbirth. Although the Nepal data are more recent (2021), they were collected during the COVID-19 pandemic, which likely heightened awareness of infection prevention. Nevertheless, compliance levels in Nepal were not substantially higher than in Kenya or Malawi, suggesting that core gaps in hand hygiene practice persist even in the context of increased attention to IPC. Given the slow pace of systemic and behavioural change in health systems, we believe these datasets remain relevant for understanding enduring challenges and informing future quality improvement efforts. We acknowledge, however, that findings should be interpreted in light of the temporal and contextual limitations of the data. More broadly, due to the cross-sectional and observational nature of our data, we cannot infer causality from the observed associations and must acknowledge the potential for bias inherent in this study design. Unmeasured factors—such as overall facility policies, workload, provider motivation, or time pressure—may influence both effective communication and HH compliance. While our findings suggest a consistent association across settings, we interpret this as a relationship that warrants further investigation rather than a definitive causal effect. Given the limitations inherent to both quantitative and secondary data analysis—particularly in capturing context, meaning, and provider–patient interactions—future research should incorporate qualitative methods to explore the underlying mechanisms influencing hand hygiene compliance, including the role of respectful communication.

In conclusion, we found low compliance with hand hygiene before clean/aseptic procedures, posing a significant risk to mothers and babies. Effective communication was consistently associated with hand hygiene compliance across countries. Further research should focus on interventions to improve compliance before aseptic procedures at birth and explore the role of respectful care in promoting hand hygiene.

## Supplementary Information

Below is the link to the electronic supplementary material.

Supplementary Material 1.

## Data Availability

The datasets analysed during the current study are available in the DHS Program repository, https://dhsprogram.com.
